# The Role of the Optical Stretcher Is Crucial in the Investigation of Cell Mechanics Regulating Cell Adhesion and Motility

**DOI:** 10.3389/fcell.2019.00184

**Published:** 2019-09-04

**Authors:** Claudia Tanja Mierke

**Affiliations:** Biological Physics Division, Peter Debye Institute for Soft Matter Physics, Faculty of Physics and Earth Sciences, Leipzig University, Leipzig, Germany

**Keywords:** cell mechanics, deformability, stiffness, viscoelasticity, cancer cells, fibroblasts, cell migration, cell adhesion

## Abstract

The mechanical properties of cells, tissues, and the surrounding extracellular matrix environment play important roles in the process of cell adhesion and migration. In physiological and pathological processes of the cells, such as wound healing and cancer, the capacity to migrate through the extracellular matrix is crucial. Hence biophysical techniques were used to determine the mechanical properties of cells that facilitate the various migratory capacities. Since the field of mechanobiology is rapidly growing, the reliable and reproducible characterization of cell mechanics is required that facilitates the adhesion and migration of cells. One of these cell mechanical techniques is the optical stretching device, which was originally developed to investigate the mechanical properties of cells, such as the deformation of single cells in suspension. After discussing the strengths and weaknesses of the technology, the latest findings in optical stretching-based cell mechanics are presented in this review. Finally, the mechanical properties of cells are correlated with their migratory potential and it is pointed out how the inhibition of biomolecules that contribute to the to the maintenance of cytoskeletal structures in cells affect their mechanical deformability.

## Introduction

The process of cell adhesion and migration has been intensively investigated in terms of genetic and molecular alterations that promote or inhibit these fundamental cellular processes. However, during the last two decades, the role of cell mechanical properties in cellular processes, such as cell adhesion and migration, has gained increasingly importance. Cells sense constantly mechanical loads and respond to them (Bao and Suresh, 2003; Buxboim et al., 2010; Janmey and Miller, 2011; Weaver, 2017) in different ways due to the force magnitude (Guolla et al., 2012) and rate (Pravincumar et al., 2012). Among these different types of forces are compressive forces applied to bone cells (Huiskes et al., 2000) shear fluid forces on vessel-lining endothelial cells (White and Frangos, 2007; Buchanan et al., 2014), and highly dynamic tensile forces within cell-layers of epithelial cells (Sumpio et al., 1987; Charras and Yap, 2018) or endothelial cells (Raupach et al., 2007; Neto et al., 2018). In response to these various forces, cells can deform vastly that evokes subsequently alterations in their biochemical phenotype. In turn, cellular forces are transmitted through a cytoskeletal clutch to a soft, three-dimensional microenvironment (Owen et al., 2017). Thereby, the coupling of the cell’s cytoskeleton to the extracellular matrix is facilitated through the translocation of vinculin and paxillin to focal adhesions that is crucial for the transmission of forces (Zhou et al., 2017). More precisely, cells seem to additionally sense neighboring cells and their mechanical properties. Beyond that, they also sense the mechanical properties of their adjacent extracellular matrix microenvironment (Engler et al., 2004). Hence, the matrix environment can on the one hand be altered by the cells and on the other hand the matrix environment can change cell mechanics (Kumar and Weaver, 2009; Wirtz et al., 2011; Humphrey et al., 2014; Chugh et al., 2017; Mierke et al., 2018; Northcott et al., 2018; Mierke, 2019).

Due to the type of stress, cells can respond differently. For example, cells adhered to a stretchable substrate can align with their polarity axis in the direction of minimal cell deformation under uniaxial stretch, which is in the direction perpendicular to the axis of strain (Buck, 1980; Moretti et al., 2004; Livne et al., 2014; Tamiello et al., 2017), whereas cells sensing fluid shear stresses can align in the flow direction (Malek and Izumo, 1996; Poduri et al., 2017). Hence, a cell is continuously or suddenly exposed to various types of forces.

An emerging and enormously growing field in mechanobiology or physical biology is investigating the physics of cancer cells. Although molecular biology has produced a wealth of results on cancer biology, it does not yet seem to be able to clearly identify the fundamental differences between malignant and benign primary tumors. Reductionist and universal approaches have been performed by biophysicists, who have asked whether alterations in cell mechanics can account for the malignant transformation of tumors (Fritsch et al., 2010).

Therefore, it has been hypothesized that distinct cellular mechanical properties, such as the deformability of cells supports the malignant and aggressive potential of cancer cells, including increased migratory potential of cells (Guck et al., 2005). In general, a hypothesis can be raised whether increased deformability of cells lead to increased migratory capacity in 3D confinements, such as extracellular matrices. There is also a contradictory hypothesis raised that questions this general hypothesis, since cancer cell types vary greatly in their biochemical and genetic phenotypes and it can also be that there exist cancer cell type specific differences, which may not allow that increased deformability of cells causes increased migration and invasion of confined 3D extracellular matrices (Jonietz, 2012; Alibert et al., 2017). Moreover, here, it can be hypothesized that there exist differences between various cell types and it cannot be hypothesized in general and cell type independent that increased deformability of cells leads to elevated migration and invasion of 3D matrix confinements. In addition, the deformability of breast cancer cells can be altered by stimulation with cytokines, such as tumor growth factor-beta (TGF-β), depending on the mechanical phenotype of the cells (Kulkarni et al., 2018).

Since cell adhesion and migration play a role in many physiological and pathological processes, the analysis and identification of the overall mechano-phenotype of cells seems to be crucial in determining the migratory potential of cells (Mierke, 2019). Alterations in the mechanical phenotype of cells can be utilized to determine the malignant and aggressive potential that is closely associated with increased migratory capacity of cells, such as cancer cells (Mierke et al., 2008a, b, 2011b; Remmerbach et al., 2009; Mierke, 2014; Fischer et al., 2017).

In addition, mechanical properties of cells are crucial for the adhesion of cells to a flat substrate and subsequently for migration (Humphries et al., 2015). In a more natural and physiological 3D microenvironment, the cell mechanical properties may become even more crucial for cell migration and invasion through the extracellular matrix microenvironment of connective tissues (Fischer et al., 2017; Jansen et al., 2017; Kunschmann et al., 2017; Mierke, 2019). Since cells in a 3D matrix environment can mechanically sense the local matrix properties of their surroundings, such as the extracellular matrix, the matrix mechanics may in turn alter the cell mechanics (Engler et al., 2004; Mierke et al., 2018). In order to cancel out the effects of the matrix mechanics on cellular mechanical properties, the mechanical properties of cells need to be analyzed in suspension, such as with the optical cell stretcher.

Hence, this review article discusses the effect of deformability of cells and their relation to cell migration and invasion through 3D confinements and tries to explain the obvious contractions in the current literature by questioning the hypothesis raised in the field regarding the universal feature of cell mechanical properties and their effect on cellular functions. Thereby, the optical cell stretcher plays a prominent role, since it can be employed to analyze the mechanical properties of nearly all types of individual cells including natural suspended and adhesive cells (Guck et al., 2000, 2001, 2005; Kunschmann et al., 2017; Mierke et al., 2017). Thereby, strengths and limitations of the optical cell stretching technique are discussed. Finally, it is ruled out what impact the optical cell stretching device currently has and what future role in biophysical research it will fulfill.

## Cell Mechanical Probing Techniques and Adhesive State of a Cell

Besides the optical stretcher, there exist many cell mechanical probing techniques. These techniques analyze the mechanical properties of the cells by exerting active forces on them or by assessing the fluctuations of beads or the deformation of surrounding extracellular biopolymer-based matrices, such as collagen gels, or synthetic purely elastic materials, such as polyacrylamide gels (PAA). Both, active and passive cell mechanical probing techniques mostly require the direct contact between the cell and the device. Two of the force probing techniques, optical stretching and magnetic tweezer have become remarkable biophysical probing techniques for cell mechanical analysis. In optical cell stretching the cell is trapped and stretched, but no direct contact between the cell and the device is needed and hence the measurement is contactless. In contrast, for magnetic tweezer-based analysis, the contact between the superparamagnetic bead, which is generally coated with an extracellular matrix protein, and the cell is required.

In general, the process of cell adhesion plays a major role in providing the mechanical phenotype of cells and hence differences in cell adhesion can easily alter the overall mechanical properties of cells such as cell deformability (or invers the stiffness) and contractile force exertion (Mierke et al., 2008a, b). Since, cell mechanical stretching with the optical stretcher involves the non-adhesive state of the cell, the measured mechanical properties may be altered in a more physiological environment, where the cells adhered to. Thus, what role play the cell mechanical properties, when a cell is in its non-adhesive state in a non-physiological environment? Moreover, a major question can be raised whether the cell adhesive state and cell deformability are required to be decoupled in order to measure only the structural effect of the cell cytoskeleton on cell mechanics. An advantage of the optical cell stretcher is that the contribution of the cell cytoskeleton on cell mechanics can in fact be determined without the effect of cell adhesion that in turn can alter cell mechanics.

In contrast, in several cell mechanical probing techniques, such as magnetic tweezers, AFM, traction force analysis and displacement field analysis, the adherence state of a cell plays a prominent role in determining the mechanical phenotype of cells. Moreover, the adhesive state of the cell is required to perform magnetic tweezers, traction force analysis and displacement field analyses. An exception represents the AFM-based cell mechanics probing technique, since here adhesive and non-adhesive cells can be measured (Fischer et al., 2017). Moreover, when measuring adhesive cells, the contribution of the cell adhesion to cell mechanical properties cannot be discriminated from the contribution of cytoskeletal proteins and structures. Hence, the optical cell stretching techniques enables us to address cell mechanical properties of non-adhesive (suspended) cells and hence to decipher the contribution to the cell mechanical properties of cell cytoskeletal and cell organelles independently of cell adhesion-depend effects.

### Active Cell Mechanical Analysis From a Biophysical Point-of-View

**FIGURE 1:**
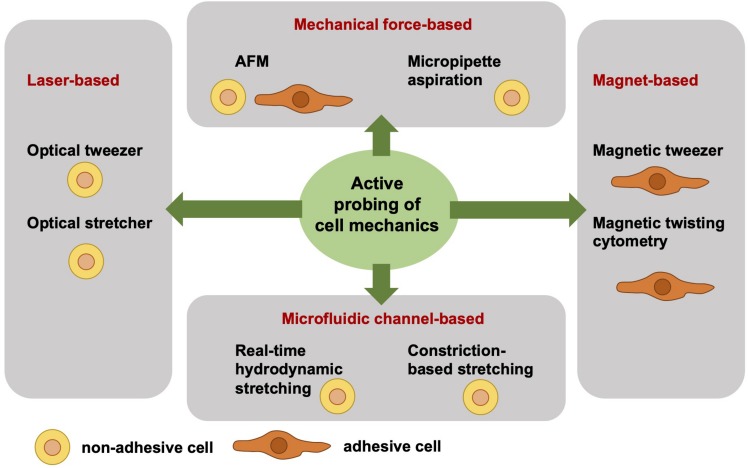
Overview of selected different actively cell mechanics probing techniques from a biophysical point-of-view. The active cell mechanical probing techniques can be subdivided into four groups, such as laser-based, mechanical forces-based, magnet-based, and microfluidic channel-based techniques. The laser-based techniques, such as optical tweezer and optical stretcher, require non-adhesive cells. Similarly, the microfluidic channel-based techniques require non-adhesive cells, such as real-time hydrodynamics stretching and constriction-based stretching. In contrast to the magnet-based techniques, such as magnetic tweezer and magnetic twisting cytometry, are performed with adhesive cells. The mechanical force-based techniques, such as AFM, can measure both adhesive and non-adhesive cells, whereas the micropipette aspiration technique requires non-adhesive cells.

Independent of the adhesive state of a cell, the cell mechanical techniques can be grouped. Active cell mechanical analysis from a biophysical point-of-view means that active microrheological techniques deliver detailed and sometimes even local viscoelastic material properties of cells. The active and direct measurement of the cell mechanics employs a well-defined force application and a precise acquisition and analysis of the resulting deformation. However, active cell mechanics does not mean that the cell is actively contracting or dynamically remodeling its mechanical phenotype. Among the active mechanical probing cell mechanical techniques are atomic force microscopy (AFM) (Gavara and Chadwick, 2012, 2016; Lekka et al., 2012; Rother et al., 2014; Haase and Pelling, 2015; Lekka, 2016; Lekka and Pabijan, 2019), optical tweezer (Ashkin, 1992; Falleroni et al., 2018), optical stretcher (Guck et al., 2000, 2001, 2005), magnetic tweezer (Kollmannsberger and Fabry, 2007; Mierke et al., 2008a, 2011b, 2017; Kollmannsberger et al., 2011), magnetic twisting cytometry (Fabry et al., 2001; Puig-De-Morales et al., 2001; Mierke, 2011a), real-time hydrodynamic stretching (Huber et al., 2018), constriction-based stretching (Gossett et al., 2012; Dudani et al., 2013; Lange et al., 2015, 2017), and micropipette aspiration (Oh et al., 2012; Lee and Liu, 2015; Figure 1). All these techniques are frequently used in the field of cell mechanics and each technique has its advantages and disadvantages compared to the other. However, the major difference between these active mechanical probing devices is the requirement of adhesive or non-adhesive (suspended) cells for the measurement. More precisely, the optical stretcher, optical tweezer, hydrodynamics stretching or confinement-based stretching and partly AFM need non-adhesive cells during the measurement. AFM measurements are performed mainly with adhesive cells. Magnetic tweezer and magnetic twisting cytometry require both adhesive cells. In some cell mechanics techniques, such as for magnetic and optical tweezers and magnetic twisting cytometry, the coupling of bead markers to the cells is required for the measurement. In general, the beads are coated with extracellular matrix proteins that bind to cell surface receptors, such as the integrin cell-matrix receptors.

There is a major difference of the cell shape between adhesive and non-adhesive cells, because non-adhesive cells exhibit a rounded symmetric shape, whereas adhesive cells can exhibit all types of shape ranging from rounded to polarized (asymmetric shapes). Hence the variation of the cellular shape may be larger between adhesive cells compared to non-adhesive cells, since non-adhesive cells uniformly adapt a symmetric shape. In order to obtain reliable and reproducible data, higher cell numbers need to be analyzed, when adhesive cells of highly altered cell shapes and polarities are measured. Commonly, these adhesion-based mechanical probing techniques are of less throughput and less automated compared to optical, hydrodynamics and confinement-based stretching and hence the number of analyzed cells is in general still lower. An advantage of the adhesive cell-based techniques is that they take cell adhesion into account, when determining cell mechanics.

### Passive Cell Mechanical Analysis From a Biophysical Point-of-View

**FIGURE 2:**
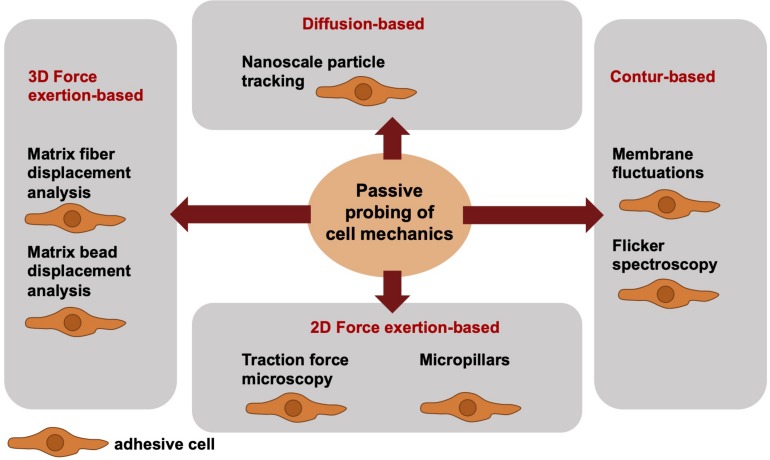
Overview of selected different passively cell mechanics probing techniques from a biophysical point-of-view. The passive cell mechanical probing techniques can be subdivided into four groups, such as diffusion-based techniques including nanoscale particle tracking, contour-based techniques including membrane fluctuations and flicker spectroscopy, 2D force exertion-based techniques including traction force microscopy and micropillars, and 3D force exertion-based techniques including matrix fiber displacement analysis and matrix bead displacement analysis. All these techniques require adhesive cells.

When addressing the passive cell mechanical techniques, the cells can be analyzed only in direct contact to the measurement device. Hence, the cells are in their adhesive state, when their mechanical phenotype is determined. More precisely, passive cell mechanical analyses are performed in the absence of well-defined local forces and analyze the fluctuations of particles or structural elements or the deformation of materials by cells. Among these techniques are the nanoscale particle tracking (Bursac et al., 2005; Mierke, 2011a), membrane fluctuation measurements or flicker spectroscopy (bending stiffness) (Döbereiner et al., 2003; Loftus et al., 2013), traction force measurements on PAA gels (Mierke et al., 2008a, b, 2011a,b) or micropillars (Heil and Spatz, 2010; Schoen et al., 2010) and matrix displacement analysis (Fischer et al., 2017; Kunschmann et al., 2019) or matrix bead displacement analysis (Franck et al., 2011; Steinwachs et al., 2016; Cóndor et al., 2017), when cells themselves migrate and invade through a 3D confined extracellular matrix (Figure 2). The limitations associated with mechanical techniques pose challenges to the detection of high-resolution spatiotemporal alterations of cells or cell compartments. Particle tracking of intracellular nanometer-sized beads has been employed to confirm a connection between intracellular regulation (cytoskeletal remodeling processes) and stiffening to cell motility and subsequently to perturbed mechanotransduction in cancer cells, such as breast cancer cells. Moreover, it may be hypothesized that the adaptation of intracellular contractility and stiffness are dependent on the mechanical properties of the extracellular matrix environment, such as matrix stiffness.

## The Optical Cell Stretching Principle

**FIGURE 3:**
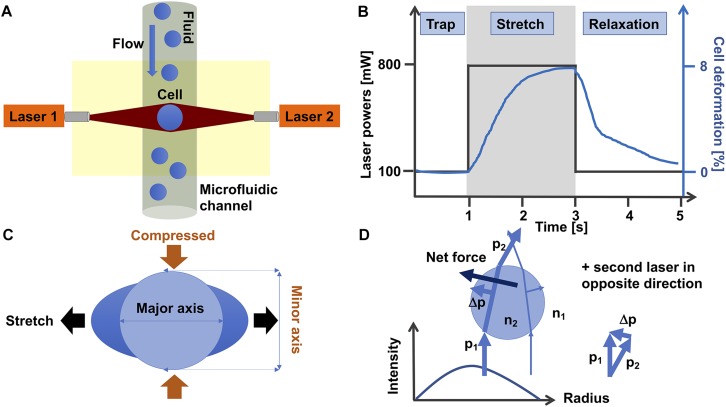
Optical cell stretching technique. **(A)** Single non-adhesive cells are transported by flow in a microfluidic system that is placed perpendicular to two opposite divergent non-focused laser-beams and can trap and stretch individual cells one after another. **(B)** A standard protocol for a cell stretching experiment is as follows: Firstly, a cell is trapped for 1 s with 100 mW laser power at each laser. Secondly, the cell is stretched with 800 mW laser powers for 2 s. Thirdly, the cell is relaxed by switching the high laser powers to 100 mW (trapping laser powers) for 2 s. The black line shows the laser power profile and the blue line the deformation of the cell. **(C)** During the stretching phase, the cell is stretched at its major axis (parallel to the laser beam axis) and compressed at its minor axis (perpendicular to the laser beam axis). **(D)** The momentum of two rays with different intensities show how these forces propagate through a cell. The intensity profile of the laser is given in the diagram. For simplicity, the second laser in the opposite direction is omitted. The blue spherical object represents a single cell.

The optical stretching device is composed of two laser beams that are positioned face to face and have usually a wavelength of 1064 nm that lies in the infrared spectrum (Figure 3A). At this wavelength, cell damage due to heating can usually be prevented. In line with this, the laser beams are not focused, which reduces the damage to the cells. More precisely, the optical stretcher is a double beam trap in which two slightly diverging laser beams with a Gaussian profile are able to trap a cell in the middle of the beams. The laser beams are positioned perpendicular to an optical flow chamber through which the single spherical cells are transported by a microfluidic pump system. The cell stabilization is given, since the total force on the cell is zero. This condition can be reached, when the refractive index of the cell is larger than the refractive index of the cell surrounding fluid. The laser beam size must be larger than the cell size.

The cell is trapped for 1 s with the two lasers, each running at 100 mW laser power (Figure 3B). When the cell is trapped, the microfluidic flow is switched off and the laser powers are increased to 500–1200 mW in order to deform the cells at their cell axis parallel to the laser beams axis for 2 s. More precisely, the momentum transfer happens at the cell surface, where the net force that acts on the entire cell is zero (Guck et al., 2001), which is provided by the symmetric geometry of the double-beam trap. When the cell is sufficiently elastic, the two laser beams can deform the cell by stretching it along the laser beam axis.

For each cell type the “optimal” stretching power needs to be determined by using various laser stretching powers. After returning the laser powers to the trapping laser powers, the relaxation of the cell is recorded for at least 2 s. The cell is stretched along its major axis parallel to the laser beam axis and contracted along its minor axis perpendicular to the laser beam axis (Figure 3C). However, there are even cells that behave differently, as they are not much contracted at their minor axis, when stretched along their major axis (Kunschmann et al., 2017). It has been revealed that all cells exhibit viscoelastic behavior (Guck et al., 2001, 2005; Kunschmann et al., 2017, 2019; Mierke et al., 2017).

The general principle of the laser-based optical traps is that momentum is transferred from the laser light toward the cell. Following Newton’s second law, the momentum transfer to the cell, in turn exerts a stretching force on the cell (Figure 3D). In summary, this device can stretch various dielectric materials, including cells, and thereby their viscoelastic properties can be determined fast and without any direct influence on the cell. The optimal light intensity is given, when the laser diameter is slightly larger than the cell diameter.

Taken together, the cell stretching force depends on the relative index of refraction *n* and on the ratio of the cell radius and the laser beam radius. The smaller the laser beam radius, the more intense the light propagating through the cell and the more stress is exerted on the cell surface. When the ratio between the beam radius and the cell radius is smaller than 1, the trapping of the cell is unstable. The optimal trapping is achieved when this ratio is slightly larger than 1, since the calculated stress profile approximation corresponds almost exactly to the true profile (Guck et al., 2001).

In order to fulfill the ray-optics regime condition, the cell diameter needs to be larger than the laser wavelength. In this regime, no distinction between reflection, refraction and diffraction components is required. Moreover, the perturbation of the incident wavefront is relatively small, the cell can be treated as an induced dipole that underlies simple electromagnetic laws. Hence there are two forces acting on the cell, such as a scatter force parallel to the laser beam axes and a gradient force perpendicular to the scatter force. The gradient force arises due to the Lorenz force that acts on the cell dipole, which is induced by the electromagnetic field. Since, the two lasers face each other, the scatter forces cancel out and only the gradient forces remain. The gradient forces are toward the highest intensity of the laser beam axes.

The incident laser beams are decomposed into individual rays that possess a distinct direction, intensity and momentum. All rays propagate in a straight line, when they are in uniform and non-dispersive matter, such as cells, and hence geometrical optics can be applied to describe them (Figure 3D). When a light ray has traveled through the cell, the ray momentum is altered in magnitude and direction. This difference in momentum is transferred to the cell. All net forces are applied to the cell surface and hence a soft object, such as a cell, is deformed.

## Strengths of the Optical Cell Stretching Technique

The major strength of the optical cell stretcher is its applicability to a wide range of cell types in their non-adhesive state. Thereby, the cells can be measured in the presence or absence of pharmacological drugs probing cytoskeletal proteins, adaptor proteins, or mechanotransductive proteins. Among these cell types can be naturally suspended and adherent cells of established cell lines and additionally primary cell cultures can be analyzed. Besides homogeneous cell populations, heterogenous cell populations can be analyzed and major subpopulations can be identified based on their mechanical phenotype such as cell deformation along the laser beam axis and cell retraction of the perpendicular cell axis. Besides the deformation behavior upon stretch, the relaxation behavior of the cells can be monitored after removal of the stretching force. Although the optical stretching technique allows a higher and hence intermediate throughput of cells that are optically stretched, it is far away from a high throughput technique. There are hydrodynamics or confinement-based microfluidic techniques available that can analyze thousands of cells per minute (Lange et al., 2015, 2017). Moreover, these relatively high throughput techniques can analyze the cells in real time and thereby still reach analysis rates of 1000 cells per second (Huber et al., 2018).

A major advantage of the optical stretching technique is that the whole cell mechanical properties can be determined quantitatively at intermediate-throughput and independently of the user. All cells, which flow through the measurement microfluidic channel, can generally be tracked and measured, when the cell concentration in the sample fluid volume is appropriate. The bulk cellular mechanical properties can be determined at the single cell level and hence the elastic and viscous behavior of different cell types can be revealed. In addition to the behavior of the cells upon stress, the relaxation behavior of the cells can be analyzed. As an alternative variant of the force (stress) application approach with the optical stretcher, the force (stress) application can be repeated and also increased in its strength to probe also stress stiffening or stress softening behavior of the cells. Finally, the optical cell stretching technique enables us to the measure the mechanical properties of different cell types under standardized conditions. Hence, cell types with different adhesive capacities can be compared in their mechanical phenotypes that are independent of the adhesion process. Besides cancer cells, these cell types may be epithelial cells, fibroblasts, and endothelial cells, which cell mechanics are altered during developmental processes, cellular differentiation or pathological condition including fibrotic-dependent diseases. In detail, alterations of cell-matrix or cell-cell adhesion receptors can be decoupled from the cellular mechanical properties, as the cells are analyzed in suspension using the optical cell stretching technology.

## Limitations of the Optical Cell Stretching Technique

A weakness of the optical cell stretching technique is that the laser wavelength should be chosen in the infra-red wavelength, where the heating of cells is increased. The heating is a major issue of the optical cell stretcher (Ebert et al., 2007), since cells may be severely damaged and undergo apoptosis (Wetzel et al., 2011), which in turn also affects their mechanical phenotype. However, a heating of 58°C will still have survival rates of 60% (Wetzel et al., 2011). Above a critical cell temperature of 52°C cell contraction due to heating has been observed (Chan et al., 2014) and the flux of calcium is altered (Gyger et al., 2011). Moreover, when measuring dynamic responses of cells over different time intervals and comparing different cell types, the time intervals need to be kept constant, since different time scales of heating impact differently on cell mechanics using the optical cell stretcher (Kießling et al., 2013).

Another major weakness or limitation of the optical cell stretcher is that certain cells, such as melanoma cells, which possess large dark particles, cannot be analyzed. When the laser beam hits these dark spots, these black surfaces absorb the laser light and subsequently the cell explodes. When this happens, the entire microfluidic setup can be destroyed and hence needs to be rebuild. Another related minor point is that several pharmacological drugs, such as the formin FH2 domain inhibitor SMIFH2, absorb near the infrared laser light and cells stimulated with this drug cannot be measured using the optical stretcher with a wavelength of 1064 nm. However, in the analysis of biological organisms, laser light near the infrared range, such as 700–1100 nm is often used in order to avoid radiation damage that occurs at shorter wavelengths. Moreover, distinct pharmacological drugs may not be suitable for optical stretcher experiments, when they affect the viability of the cells and induce programmed cell death (apoptosis). Specific cell types or cell lines, such as selected MDA-MB-231 human breast cancer cell variants, cannot be measured in the optical stretcher, since over 85% of the cells die during the measurement and hence only a small cell subset can be analyzed. However, some variants of MDA-MB-231 cells can be measured with the optical stretcher (Guck et al., 2005). Beyond established cell lines, primary cells, such as human microvascular endothelial cells can be analyzed in principle with the optical stretcher, however, a majority of the cells in the cell population may die, since they are required to be in a non-adhesive state that renders them to undergo apoptosis. In summary, the cell viability is crucial for the determination of the cell mechanical properties and therefore needs to be precisely controlled. Another weakness of this optical stretching technique is that only cells of nearly equal cell sizes can be compared. Moreover, the cell sizes of pharmacological drug treated cells, which are compared using the optical stretching technique, are required to be in the same range during treatment (Kubitschke et al., 2017; Kunschmann et al., 2019).

In contrast to other biophysical methods, the optical stretcher requires spherical objects, such as suspended cells, which are not exerting large protrusions on their cell membrane surface. The spherical objects are required in order to exert the same forces acting on the rounded cells. Hence, a ray-optics approach can be employed to calculate the optical stress distribution and the net stretching force. When these cells exert protrusion, the force distribution on the cell cannot be calculated precisely (Guck et al., 2001, 2005). However, most cell types, such as adherent and suspended cells, can be measured. The detection algorithm has been refined and detects also cells with small deviations from spheres, as it is usually the case for non-adhesive and hence suspended cells. It can be noted that the cells are measured in a rather non-physiological environment and buffer conditions can alter cell mechanical properties, such as cell deformation. Thus, the conditions of the measured cell types need to be kept the same to reduce variations caused by experimental side effects. Finally, during the entire measurement time, such as several hours, the non-adhesive cells need to maintain their spherical shape and stay viable. For each cell type the duration of the measurement time need to be determined to exclude effects on cell deformation caused by cell death.

Although the optical cell stretching technique is an intermediate-throughput approach, other microfluidic techniques based on hydrodynamics or constriction can achieve at least twofold increased cell numbers per minute to several thousands of cells per minute. In order to increase the number of cells that are measured per minute, knowledge of the optical stretching technique is required to improve the analysis process and the cell preparation, where cell clustering, cell mucus and dead cells need to be avoided. However, under normal laboratory conditions, the limit is reached at approximately 3–6 cells per minute for epithelial cells or fibroblasts.

Since the optical cell stretching technique is environmental independent, complex 3D microenvironments cannot be implemented and hence other cell mechanical techniques are still required to analyze the cell mechanical properties in dependence of specific microenvironmental cues. Indeed, these extracellular matrix environments or specific cellular environments can alter the cell mechanical properties (Engler et al., 2004; Discher et al., 2005; Mierke et al., 2008a, 2018).

## Role of the Optical Stretcher in Cell Mechanics Controlling Cell Migration

**FIGURE 4:**
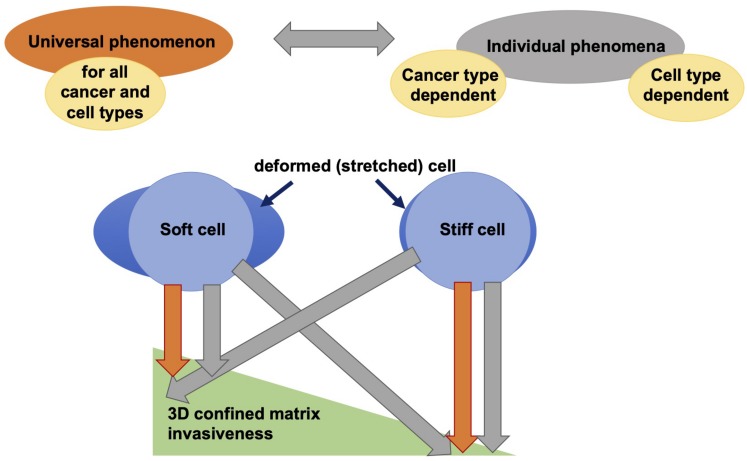
Is the correlation between deformability and cell invasiveness in 3D confined microenvironments a universal or an individual phenomenon? There is still a contradictory discussion about the relationship between cell deformability and cell motility through 3D confinements. One hypothesis states that this relationship is universally applicable to all cancers (cancer types) and even to all cell types, and another contradictory hypothesis states that there are differences between cancer cells and cell types in terms of the relationship between cell stiffness (or inverse softness) and invasiveness in 3D extracellular matrix confinements.

While genes and biochemical signaling pathways provide strongly controlled cellular properties, it seems to be obvious that mechanical interactions between cells and their environment are a crucial determinant providing cellular functions such as motility that enables them to finally migrate to targeted regions during physiological and pathological processes in three-dimensional tissues. Each contact of a cell with the its environment inherently requires the mechanical interactions between cells and extracellular matrix networks or other matrix embedded cells (Mierke et al., 2008a, 2011a; Kumar and Weaver, 2009; Brábek et al., 2010; Mierke, 2011a, b, 2014; Wirtz et al., 2011; Humphrey et al., 2014; Chugh et al., 2017; Northcott et al., 2018). Besides the molecular phenotype of cells, their mechanical phenotype is hypothesized to play an important role in regulating the migration of cells through 3D extracellular matrix confinements. In order to predict how cell mechanics influences cell migration and development of tissues or diseases *in vivo*, the emerging mechanical behavior for different cell types on different length scales ranging from molecular to whole tissue level needs to be taken into account. Hence there exists no overall picture of the matrix mechanics of the cellular environment and even essential aspects of cell and tissue behavior are not clearly understood. The mechanical sensing and mechanotransduction processes are supposed to be cell type and tissue specific. In addition, since cell mechanics are defined by universal physical laws, it is hypothesized that there exists a universal behavior of cells that exhibit a specific mechanical cellular phenotype (Figure 4; Fritsch et al., 2010). Hence, cell migration in 3D environments, such as tissues, is defined by a mixture of both highly cell specific molecular events and fundamental physical properties that apply to all different kinds of cell types. In contrast to the universal applicability of the linkage between distinct mechanical properties and specific cellular functions, such as cell migration in 3D confined extracellular matrices, it has been questioned whether there exists such a universal linkage independent of the cell type (Figure 4; Jonietz, 2012; Alibert et al., 2017) and possibly also between malignant and non-malignant cancer cells. Recently, a difference in the linkage between cell stiffness (invers deformability, softness, or compliance) and motility of cells has been revealed, since stiffer fibroblasts are more invasive into artificial 3D extracellular matrix confinements (Mierke et al., 2010; Kunschmann et al., 2017, 2019). Thereby, it seems to be rather independent whether the cell mechanical properties were determined of adhesive or non-adhesive cells, since the cytoskeletal mechanical properties are still detectable and independent of the adhesive state of the cell (Fischer et al., 2017; Meinhövel et al., 2018). However, the connection between stiffness and invasiveness seems to be apparently different in breast and oral cancer cells, where the inverse has been observed. In fact, softer (compliant or more deformable) cells migrate more into artificial 3D extracellular matrix confinements (Guck et al., 2005; Remmerbach et al., 2009; Runge et al., 2014; Meinhövel et al., 2018). All these studies deal with the optical cell stretching technique. However, it has been shown that softer cells migrate more into 3D matrices, where the cell mechanical properties have been determined with AFM (Fischer et al., 2017).

### Contribution of the Optical Stretching Technique to Cell Mechanics

In order to increase the knowledge of cell mechanical properties, it needs to be revealed what kind of molecule, its activation state or structural component contribute to the mechanical phenotype of cells. It has turned out that the phosphorylation state of cellular proteins can impact the mechanical properties of cellular compartments, regions and entire cells. An example for a candidate molecule is the focal adhesion kinase (FAK). More precisely, it has been found that FAK fulfills kinase-dependent and kinase-independent cytoskeletal scaffolding functions (Cance et al., 2013). However, the potential role of these two activities of FAK in a specific type of cancer remains largely less well understood (Gao et al., 2015). Using an optical cell stretcher, it has been shown that the stiffness of the cells is increased (Mierke et al., 2017), when the ATP-binding deficient Lys454-to-Arg mutation of FAK (R454 FAK) is expressed that lacks the kinase activity (Lim et al., 2010). This finding is in line with results showing that malignant cancer cells, which express increased FAK, can also be significantly stiffer using a magnetic tweezer device, where the cellular stiffness is determined of cells in their adhesive state (Mierke et al., 2011b). However, it cannot be excluded that the mechanotransduction signaling via FAK contributes to the malignant progression of cancer types. Using the magnetic tweezer, the displacement of superparamagnetic beads in a radial magnetic field can be determined for various cancer cells, such as MDA-MB-231 and MCF-7 human breast cancer cells. The displacement of the fibronectin coated beads coupled to integrins, such as the α5β1 integrin cell-matrix receptor, on the plasma membrane is higher in MDA-MB-231 cells compared to MCF-7 cells indicating that the MDA-MB-231 cells are softer (more compliant) compared to MCF-7 cells. Moreover, AFM measurements on the same fibroblast cell types in suspension (non-adhesive state) revealed that the FAKR454/R454 cells are stiffer compared to control cells (Mierke et al., 2017). In contrast, when these two cell types are analyzed in their adhesive state using the AFM, the FAKR454/R454 cells are softer compared to control cells. Another example is the CD97 receptor that changes the entire cellular mechanical properties, when the phosphorylation of CD97 is inhibited. More precisely, the cells displayed increased deformability and hence are softer (Hilbig et al., 2018). In line with this, cytoskeletal modifications revealed that cell adhesion plays a role in determining the cell mechanical properties (Golfier et al., 2017). Finally, cellular mechanical properties and fluorescence signals of cells that overexpress the GFP-tagged nuclear envelope protein lamin A, have both been determined simultaneously. Thereby, a dose-dependent increase of the cell elastic modulus and a decrease of cellular fluidity have been observed, when lamin A levels are increased (Lange et al., 2017).

### Correlation of Cell Deformability With Their Motility

During the last two decades, it has been found repeatedly that the invasiveness and aggressiveness of cells correlates with their deformability (inverse of stiffness) (Guck et al., 2001, 2005; Remmerbach et al., 2009; Fritsch et al., 2010; Mierke et al., 2010, 2011b, 2017; Runge et al., 2014; Fischer et al., 2017; Kunschmann et al., 2017, 2019; Meinhövel et al., 2018). In general, it has been questioned whether there exists a simplistic universal connection between cell mechanics, such as deformability, and cell invasion into 3D microenvironments. More precisely, can it be stated in general in a very simple manner that the more deformable cancer cell lines, primary cancer cells and fibroblasts are highly invasive compared to less deformable control cells, which are only weakly or non-invasive?

Indeed, several *in vitro* studies dealing with cancer cell lines of breast and cervix carcinomas have demonstrated that the viscoelastic properties of normal and healthy cells are pronouncedly different compared to malignant cancer cells (Beil et al., 2003; Guck et al., 2005; Fritsch et al., 2010). In detail, cancer cells can undergo a neoplastic transformation that is associated by an overall decrease of the cytoskeletal polymers, such as actin and actin-interacting proteins, and thereby alters the structural integrity of the entire cell (Rao and Cohen, 1991; Ananthakrishnan et al., 2006; Hall, 2009; Efremov et al., 2014). A pilot study of established cancer cell lines of the oral cavity is in line with these findings, since these cancer cell lines, which are analyzed with an optical stretcher device, display uniformly that more deformable cells are more aggressive (Remmerbach et al., 2009). However, it has to be addressed that also the environment of the cancer cells, such as extracellular matrix and embedded cells affect their mechanical properties (Kumar and Weaver, 2009; Mierke, 2011a, b, 2019; Wirtz et al., 2011; Buchanan et al., 2014; Humphrey et al., 2014; Chugh et al., 2017; Mierke et al., 2018; Northcott et al., 2018). In addition, it has been hypothesized that the phenomenon of the mechanical property alterations in cancer cells during the progression of cancer malignancy can be employed to identify and diagnose cancer and predict the disease state. The simple hypothesis needs to be connected to other biochemical or genomic/proteomic approaches in order to create a tool for determining the efficiency of a certain drug or treatment. However, a reliable pre-selection of drugs can be made by focusing on the mechanical phenotype, which is highly influenced by the microenvironment and matrix material properties.

In fact, improvement of optical stretching technology showed that the cellular deformability depends additionally significantly on the conditions for the cell culture and pre-treatments before and during the assessment of cell mechanics (Runge et al., 2014). Indeed, it has been revealed that the deformability of cells can change with the temperature during the cell stretching procedure (Kießling et al., 2013; Warmt et al., 2014; Schmidt et al., 2015). However, it has been found that healthy and malignant oral tissues can be distinguished based on altered cell mechanical properties, such as different deformation and relaxation behavior of the cells (Meinhövel et al., 2018). More precisely, it can be hypothesized in a simple manner that the softening of the cancer cells is a prerequisite for their malignant progression and hence the softness of cells can be used as a universal marker for the cancer cell aggressiveness. Subsequently, the metastatic potential of the entire tumor can be predicted by analyzing the deformability of individual cancer cells derived from the primary tumor. However, it is still under question, whether it can be applied for all cancer types and without the effect of the microenvironment.

Besides these obvious findings, there is still a controversial discussion about the softness or stiffness and its universal contribution to the malignant state of cancer cells. Several times, it has been reported for distinct cancer types that malignant and invasive cancer cells are softer (Beil et al., 2003; Guck et al., 2005; Remmerbach et al., 2009; Fritsch et al., 2010; Meinhövel et al., 2018), whereas other studies of specific cancer cell types showed that malignant and invasive cells are stiffer compared to healthy or less invasive cells (Mierke et al., 2008a, 2011a,b; Mierke, 2013). These contradictory results may question the aforementioned universal hypothesis that malignant cancer cells need to be softer compared to non-malignant cancer cells or healthy cells of the same cell type. Hence, it is not clearly understood whether all different cancer cell types should display the same cell mechanical properties, such as softness (compliance or inverse stiffness), as a universal feature or whether the cell mechanical properties depend on the cancer cell type (Jonietz, 2012; Alibert et al., 2017). However, there is agreement that the malignancy of cancer cells can be predicted by determining their cell mechanical properties, such as deformability (inverse stiffness) (Beil et al., 2003; Guck et al., 2005; Mierke et al., 2008a, 2011a,b; Remmerbach et al., 2009; Fritsch et al., 2010; Mierke, 2013; Meinhövel et al., 2018) and/or the contractile force exertion of cells to their local microenvironment (Fischer et al., 2017).

The optical cell stretcher has still the potential to serve as an intermediate-throughput diagnostic device, as it has been shown for certain cancer type such as breast (Guck et al., 2005; Li et al., 2008) and oral (Remmerbach et al., 2009) cancers. In detail, it has been demonstrated that alterations in the optical deformability of certain cancer cells can be an indicator for the malignancy of cancer cells and the overall metastatic potential for distinct cancer types. Since cancer cells display elevated proliferation, it still needs to be analyzed whether effects of the cell-cycle and enhanced proliferation generally affect cell deformability. When using a heterogenic cell population isolated from a primary tumor, it may also contain other cells than cancer cells, such as stroma cells or endothelial cells that are even stimulated by inflammatory cytokines. Since different cell types may possess a different cell deformability, additional immunostainings with fluorescent antibodies may help to distinguish between inflammatory cells of the surrounding tumor stroma and cancer cells. The inflammatory stimulation of neighboring cells, such as stroma cells, by cancer cells of the primary tumor is known (Mierke et al., 2018) and may alter the mechanical deformability of stroma cells similar to cancer cells and hence may have an impact on the identification of malignant and metastatic cancer cells. When the cell mechanical properties can serve as an indicator for the malignancy of tumors, cell mechanical measurements can also be used to determine the efficacy of distinct pharmacological anti-cancer drugs (Suresh, 2007), such as cytochalasins, vinca alkaloids, and taxanes that all can affect the cytoskeletal architecture by altering cellular mechanical properties (Lam et al., 2007; Fletcher and Mullins, 2010) and thereby impairing cancer cell proliferation (Jordan and Wilson, 1998; Zhou and Giannakakou, 2005; Di Carlo, 2012).

## Concluding Remarks and Outlook

As a future perspective, I expect increased technological progresses in the field of cell mechanics that will promote the advancements and even development of novel techniques. All of which will lead to an extended range of applicable and measurable forces with possibly an improved spatial-temporal resolution. The optical cell stretcher device is a biophysical tool that can be employed to measure a broad variety of different cell types in their non-adhesive state. The stretched cell types range from individual cells that grow in suspension to those that require adhesion to their surrounding microenvironment. Besides established cell lines, primary cells derived from various organs can be measured directedly after their enzymatic or mechanical isolation from tissue resections or biopsies. Moreover, it is possible to measure small symmetrical clusters of cells, such as spheroids, however, the microfluidic channels need to be enlarged that the spheroids can be transported without being caught between the two channel walls. Since the neighboring cells seem to be important for the regulation of cell mechanical properties, cell spheroids seem to be an advanced option to determine bulk cell mechanical properties in a more physiological environment and in an adhesive state of the cells.

The optical cell stretching technique has revealed major insights into the field of cell mechanics, as the elastic and viscous behaviors of many cell types have been explored and these findings have been connected to diseases such as cancer (Guck et al., 2005; Li et al., 2008; Remmerbach et al., 2009) or other pathological or developmental processes, such as aging (senescence) of tissues (Schulze et al., 2010). Apart from whole cell mechanical properties, the mechanical properties of cellular compartments, such as the nucleus, can be measured. The mechanical characterization of the cell nucleus is important and requires more research effort, since the processes regulating the mechanotransduction in the nucleoskeleton are facilitating cellular functions (Dupont et al., 2011; Tajik et al., 2016; Croci et al., 2017). The nuclear mechanics can be assessed using the optical stretcher, as the nucleus is also stretched when the cell is stretched, and the nuclear deformation may be easily assessed by improving the computational analysis process.

Finally, the focus of future cell mechanics research is the combination of different biophysical tools in order to reveal the impact of certain effects on the whole mechanical phenotype of cells. This task seems to be a major future breakthrough in cell mechanics and includes the transformation of mechanical properties analysis to 3D microenvironments. Thereby, the pre-sorting of mechanical phenotypes still requires the optical cell stretching device, as it can deliver cell and compartment mechanical phenotypes for single cells independent of cell adhesion processes. In future applications, a combination of traditional molecular markers, such as biomarkers, based on fluorescence and label-free cell mechanical properties may further increase the functional connection of cell mechanics and the presence of distinct molecules including their localization. Hence, this combination will help to shed new light onto the underlying principles that cells employ to move or interact inside tissues.

## Author Contributions

The author wrote the entire manuscript and prepared all the figures.

## Conflict of Interest Statement

The authors declare that the research was conducted in the absence of any commercial or financial relationships that could be construed as a potential conflict of interest.
